# Advances in Multiple Sclerosis 2017

**DOI:** 10.3390/ijms19030901

**Published:** 2018-03-19

**Authors:** Kerstin Göbel, Christoph Kleinschnitz, Sven G. Meuth

**Affiliations:** 1 Department of Neurology, University of Münster, 48149 Münster, Germany; sven.meuth@ukmuenster.de; 2 Clinic for Neurology, Essen University Hospital, 45147 Essen, Germany; christoph.kleinschnitz@uk-essen.de

Multiple sclerosis (MS) is one of the most emerging fields in neurology. Recent pathophysiological insights have cleared the way for novel therapeutic strategies to combat disease aggravation. For instance, the B-cell depleting antibody ocrelizumab was recently approved for the treatment of relapsing-remitting (RRMS) and early primary progressive MS (PPMS). However, despite this progress, unmet medical necessities remain, especially for pediatric MS. Both new and more sophisticated therapeutic strategies and improved markers for predicting disease progression and/or treatment responses are still in demand.

In the special issue “Advances in Multiple Sclerosis 2017”, promising findings of this most prevalent neuroimmunological disease are depicted, covering different aspects of disease development and prediction, treatment options, and pathophysiological insights derived from both animal models and clinical studies.

For instance, the search for (novel) biomarkers predicting disease progression and/or conversion from clinically isolated syndrome (CIS) to MS is still ongoing. Schwenkenbecher et al. recently described the critical role of intrathecal IgG synthesis and visual evoked potentials (VEPs) in this context [1]. If CIS patients presented unique oligoclonal bands in the cerebrospinal fluid (CSF), conversion to MS was more than twice as likely. While pathologic VEPs did not add further information to the conversion rate by patients with oligoclonal bands, they helped to predict the conversion rate in patients with optic neuritis and absent oligoclonal bands. To avoid or at least delay conversion from CIS to MS, an appropriate therapy must be chosen. Until today, there is very little known about how responsiveness to treatment can be predicted. One small current study aimed to identify the blood signature of CIS patients to provide clues for potential immunomodulatory therapies; increased levels of transitional B cells were found in CIS patients compared to healthy donors [2]. This small study emphasizes the possible important role of B cells not only for individuals suffering from CIS, but also from MS.

Lehmann-Horn et al. recently reviewed the current knowledge of B cells in MS, including also their regulatory functions [3]. Nonetheless, further studies are needed to harness the evolving knowledge to improve the future efficacy and even safety of B cell-directed treatments. While the relevance of B cell-directed therapies in MS has to be seen in the next years, a clear strategy to increase the safety of highly effective therapy with natalizumab is still under development. There is growing concern regarding the risk of progressive multifocal leukoencephalopathy (PML), particularly after 24 doses and in patients that have previously received immunosuppressive drugs [4]. Nonetheless, clinical experiences are not homogenous, and there are no generally established guidelines so far. The same thing is true for the treatment of relapses following therapeutic plasma exchange as a second-line after a glucosteroid-unresponsive relapse. A clinical retrospective multicenter study by Ehler et al. analysed the clinical response to glucocorticosteroids for patients who developed another relapse after the completion of therapeutic plasma exchange [5]. Interestingly, the clinical improvement of patients could be detected in more than 90% of patients. In this line, glucosteroids remain as a first-line treatment for relapses in patients who were formerly unresponsive to glucosteroids. While this treatment is one of the most popular, side effects constrain its use. To overcome this problem, new delivery vehicles have been developed for improved administration. The successful administration of nanoparticles has been demonstrated in models, as reviewed by Lühder et al. [6]. However, more studies and clinical trials are needed to make new delivery vehicles available for future therapy.

Another possibility of treating MS is discussed and reviewed by Dixit et al. [7]. Helminth infections have been explored in animal models and clinical trials, and the possibility to use immunomodulatory molecules secreted by helminths may offer a more defined therapeutic strategy in the future.

Animal models, especially experimental autoimmune encephalomyelitis, or in vitro models are often used to obtain a better understanding of pathophysiology and for the development of therapies. Specific pathophysiological aspects of the microsomal prostaglandin synthetase-1, the extracellular signal-regulated kinase, (−)-β-caryophyllene, and fullerenols are described in this special issue [8,9,10,11]. Furthermore, the role of monoclonal antibodies in preclinical models is systemically reviewed [12]. Improved knowledge about factors such as zinc [13] or the chemokines CCL-17 and CCL-22 [14] can help to develop new strategies for MS treatment.

This special issue offers an interesting background of MS pathophysiology and disease development and prediction from both animal models and clinical studies, and suggests strategies for the development of MS therapies.
